# Interobserver Reliability of the Endoscopic Ultrasound Criteria for the Diagnosis of Early Chronic Pancreatitis: Comparison between the 2009 and 2019 Japanese Diagnostic Criteria

**DOI:** 10.3390/diagnostics11030431

**Published:** 2021-03-03

**Authors:** Akira Yamamiya, Atsushi Irisawa, Keiichi Tominaga, Kohei Tsuchida, Takeshi Sugaya, Misako Tsunemi, Koki Hoshi, Hidehito Jinnai, Akane Yamabe, Naoya Izawa, Mari Iwasaki, Yoichi Takimoto, Akira Kanamori, Kazunori Nagashima, Takahito Minaguchi, Ken Kashima, Yasuhito Kunogi, Ai Sato, Kenichi Goda, Makoto Iijima, Yasuo Haruyama

**Affiliations:** 1 Department of Gastroenterology, Dokkyo Medical University School of Medicine, 880 Kitakobayashi Mibu, Tochigi 321-0293, Japan; akira-y@dokkyomed.ac.jp (A.Y.); tominaga@dokkyomed.ac.jp (K.T.); tsuchida@dokkyomed.ac.jp (K.T.); t-sugaya@dokkyomed.ac.jp (T.S.); tsubo@dokkyomed.ac.jp (M.T.); hoshi@dokkyomed.ac.jp (K.H.); h-jinnai@dokkyomed.ac.jp (H.J.); yamabe@dokkyomed.ac.jp (A.Y.); izawanao@dokkyomed.ac.jp (N.I.); kizuki@dokkyomed.ac.jp (M.I.); ytak@dokkyomed.ac.jp (Y.T.); k-akira@dokkyomed.ac.jp (A.K.); n-kazu@dokkyomed.ac.jp (K.N.); takahito@dokkyomed.ac.jp (T.M.); ken-k@dokkyomed.ac.jp (K.K.); ykunogi@dokkyomed.ac.jp (Y.K.); goda@dokkyomed.ac.jp (K.G.); mkiijima@dokkyomed.ac.jp (M.I.); 2 Department of Gastroenterology, Dokkyo Medical University Nikko Medical Center, 632 Takatoku Nikko, Tochigi 321-2593, Japan; a-satoh@dokkyomed.ac.jp; 3 Department of Public Health, Dokkyo Medical University School of Medicine, 880 Kitakobayashi Mibu, Tochigi 321-0293, Japan; yasuo-h@dokkyomed.ac.jp

**Keywords:** early chronic pancreatitis, interobserver reliability, endoscopic ultrasound

## Abstract

In 2009, diagnostic criteria for early chronic pancreatitis (DCECP2009) were proposed by the Japan Pancreas Society. This study aimed to evaluate the interobserver reliability (IOR) of endoscopic ultrasound (EUS) criteria for diagnosis of early chronic pancreatitis (ECP) between DCECP2009 and 2019 diagnostic criteria for ECP (DCECP2019) to assess the validity of the revision from the perspective of EUS findings. Among patients who underwent a detailed observation of the pancreas by EUS at our institution between January 2018 and March 2019, EUS images of 97 patients were extracted. Images were reviewed by 12 gastrointestinal endoscopy experts (eight pancreatologists, group A and four nonpancreatologists, group B). The overall kappa (K)-values for the IOR of the DCECP2009 EUS criteria were 0.424 and 0.563:0.231 for groups A:B, whereas the overall K-values for the DCECP2019 criteria were 0.618, and 0.733:0.442 for groups A:B. Regarding changes in the final diagnosis of ECP based on clinical features and EUS findings, 20 cases were definite ECP, 53 were probable ECP, and 24 were normal according to DCECP2009. In contrast, seven were definite ECP, 19 were probable ECP, and 71 were normal according to DECEP2019. IOR of DCECP2019 was higher than that of DCECP2009, which indicates an improvement in precision.

## 1. Introduction

Chronic pancreatitis (CP) is an irreversible and progressive inflammation of the pancreas. CP is characterized by extensive fibrosis in the pancreatic glands due to persistent and recurrent inflammation, which eventually leads to pancreatic exocrine and endocrine secretion disorders [1,2,3]. Its incidence in Japan is 52/100,000 individuals/year, which is continuing to increase [4]. CP is associated with a high incidence of pancreatic cancer, and early diagnosis and intervention can aid in preventing disease progression. In 2009, diagnostic criteria for early chronic pancreatitis (DCECP2009) were proposed by the Japan Pancreas Society [5]. The pathology and prognosis of early CP patient groups based on diagnostic criteria have been partially elucidated, but further clarification is needed [6,7]. According to DCECP2009 and DCECP2019, the diagnosis of early CP (ECP) requires Endoscopic ultrasound (EUS). The usefulness of EUSs was first reported in 1980 [8,9]. Since then, EUSs have been applied to the diagnosis and treatment of not only pancreatic diseases but also diseases of various organs [10,11,12,13,14,15]. EUSs are excellent for detecting small lesions due to their superior image resolution, and tissue sampling is also possible by applying this technique. According to DCECP2009, patients with two of four clinical signs (recurrent abdominal pain, blood and urinary pancreatic enzyme abnormalities, pancreatic exocrine disorder, and persistent heavy alcohol consumption equivalent to at least 80 g/day of pure ethanol (EtOH 80 g/day)), and those with more than two EUS-specific findings should be diagnosed with ECP (Table S1). Ten years after DCECP2009 was established, the ECP criteria were revised in 2019 (DCECP2019) [16,17]. In this revision, based on the accumulated data and expert opinion, EUS findings were grouped into confounding findings (hyperechoic foci without shadowing and stranding and lobularity with and without honeycombing), and the number decreased from seven features to four. Clinical features were also slightly modified (Table S2). DCECP2019 aimed to increase the diagnostic specificity of DCECP2009 (Tables S1 and S2). However, the interobserver reliability (IOR) of the EUS findings in the diagnosis of CP has been previously discussed, and the IOR of the EUS findings based on the Rosemont classification is not necessarily high [18,19]. We observed similar results in the literature review of the IOR of the EUS findings in the diagnosis of CP [20]. The use of diagnostic criteria that have been shown to have high IOR may lead to a highly accurate final diagnosis. Thus, regardless of the diagnostic criteria, if the IOR is high enough, the diagnostic validity cannot be disputed. DCECP2019 summarizes the findings from DCECP2009, and the IOR is expected to increase, but evidence for this has not been examined. In this study, we evaluated the IOR to determine the diagnostic accuracy of DCECP2009 and DCECP2019 in terms of EUS findings and confirmed that DCECP2019 has an increased IOR compared with DCECP2009. Finally, we evaluated the validity of the revision.

## 2. Materials and Methods

### 2.1. Study Design

This study was performed at Dokkyo Medical University and was approved by the Medical Ethics Committee of our institution (R-25-6J). The study was registered with the University Hospital Medical Information Network Clinical Trials Registry (000040676). In place of omitting informed consent, a means to opt out was provided, which allowed research subjects to be notified and enabled the publication of research information on our website.

The primary endpoint was the comparison of IOR of the DCECP2009 and DCECP2019 EUS criteria for the diagnosis of ECP. Secondary endpoints were: (1) IOR validation for each EUS finding in DCECP2009 and DCECP2019, (2) differences in kappa (K)-values between pancreatologists and nonpancreatologists, and (3) diagnostic changes due to the revised criteria (DCECP2019).

### 2.2. Observers for IOR Evaluation

EUS images were reviewed by 12 gastrointestinal endoscopy experts (board-certified fellows of the Japan Gastroenterological Endoscopy Society: A.I., K.G., A.S., K.To., K.Ts., T.S., A.Yamab., K.H., N.I., M.I., A.K., and T.M.), while A.Yamam, selected the EUS images. Of the 12 experts, eight were pancreatologists (group A) and four were nonpancreatologists (group B) based on DCECP2009 and DCECP2019. A pancreatologist was defined as an expert who has been engaged in clinical practice with expertise in pancreatic diseases for at least 5 years. Nonpancreatologists were defined as experts who have not been engaged in clinical practice with expertise in pancreatic disease for more than 5 years, but they have the skills and ability to interpret EUS images.

### 2.3. Collection of EUS Images and IOR Evaluation

**Figure 1:**
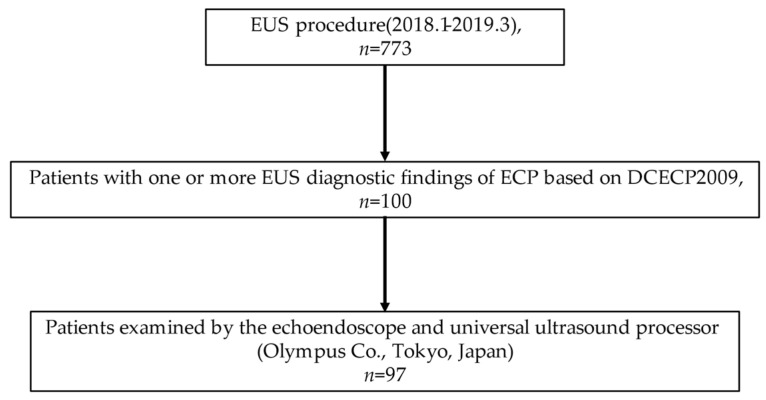
Flow diagram of this study.EUS, endoscopic ultrasound; ECP, early chronic pancreatitis.

In all, 773 patients underwent detailed observation of the pancreas by EUS at our institution between January 2018 and March 2019. When patients were diagnosed, all cases were finalized by an expert. One hundred consecutive patients with one or more EUS diagnostic findings of ECP based on DCECP2009 were extracted from the medical records by A.Ym. The examinations were performed using the GF-UE290 and GF-UCT260 (Olympus Co., Tokyo, Japan) and EG580UR and EG580UT (Fuji Film Co., Tokyo, Japan) electronic scanners. Therefore, in this study, 97 patients who were examined by GF-UE290 and GF-UCT260 were selected (Figure 1). According to the Rosemont criteria, three EUS images of the pancreatic body and tail were extracted per case [21]. Patients with pancreatic neoplasms, definite CP, and probable CP based on DCECP2009 and DCECP2019 at the time of diagnosis were excluded [5,8].

To first evaluate the IOR for each EUS finding of ECP, EUS images representing the following ECP findings were randomly selected: parenchymal features, such as lobularity with honeycombing, lobularity without honeycombing, hyperechoic foci without shadowing, stranding, and cysts, as well as ductal features, such as hyperechoic main pancreatic duct (MPD) margins and dilated side branches. EUS images of normal pancreas were also reviewed. The presence or absence of findings was evaluated by examiners who were blinded to the conditions. Each EUS finding was analyzed for IOR (multiple K statistics). Next, to evaluate the IOR of DCECP2009 and DCECP2019, three images were selected from each of the 97 selected cases, and a collection of images for evaluation was created for each case. Cases were diagnosed as ECP by observers according to the DCECP2009 and DCECP2019 and were analyzed for IOR. The standard of EUS diagnostic findings was agreed upon by most pancreatologists with the exception of A.Ym, who selected the EUS images.

### 2.4. DCECP2009

The definition of DCECP2009 is described in Supplementary Table S1. Clinical findings include (1) recurrent upper abdominal pain, (2) abnormal pancreatic enzyme levels in the serum or urine, (3) abnormal pancreatic exocrine function, and (4) continuous heavy alcohol consumption equivalent to at least 80 g/day of pure ethanol (EtOH 80 g/day). ECP is defined when a case has two or more features from (1) to (4) and when imaging findings of ECP are confirmed. Cases with only one finding (either (1) or (2)) and those that exhibit findings indicative of ECP are diagnosed with probable ECP.

**Figure 2:**
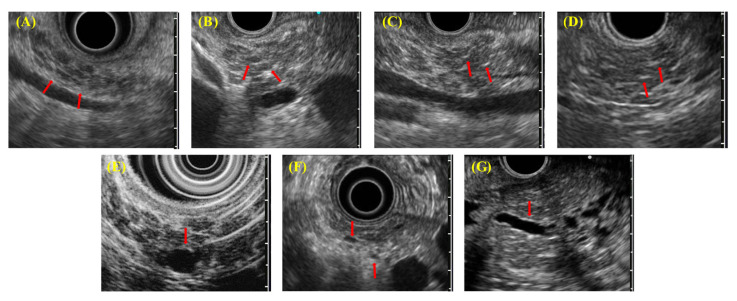
Each endoscopic ultrasound (EUS) finding in diagnostic criteria for early chronic pancreatitis 2009 (DCECP2009). (**A**) Lobularity with honeycombing; (**B**) lobularity without honeycombing; (**C**) hyperechoic foci without shadowing; (**D**) stranding; (**E**) cysts; (**F**) dilated side branches; (**G**) hyperechoic main pancreatic duct (MPD) margins.

EUS diagnostic findings (Figure 2) include (A) lobularity with honeycombing, (B) lobularity without honeycombing, (C) hyperechoic foci without shadowing, (D) stranding, (E) cysts, (F) dilated side branches, and (G) hyperechoic MPD margins. ECP is diagnosed by imaging when two or more findings, including any feature listed from (A) to (D), are observed. In addition, irregular dilatation in three or more branched pancreatic ducts, as seen by endoscopic retrograde cholangiopancreatography (ERCP), also served as imaging findings for ECP.

### 2.5. DCECP2019

The definition of DCECP2009 is described in Supplementary Table S2. Clinical findings include (1) recurrent upper abdominal pain or back pain, (2) abnormal pancreatic enzyme levels in the serum or urine, (3) abnormal pancreatic exocrine function, (4) continuous heavy alcohol consumption equivalent to at least 60 g/day of pure EtOH or pancreatitis-related genetic factors associated with susceptibility, and (5) a history of acute pancreatitis (AP). ECP is defined when three or more features from (1) to (5) are observed and when imaging findings of ECP are confirmed. Cases with two findings from (1) to (5) and that show the findings of ECP are diagnosed with probable ECP.

**Figure 3:**
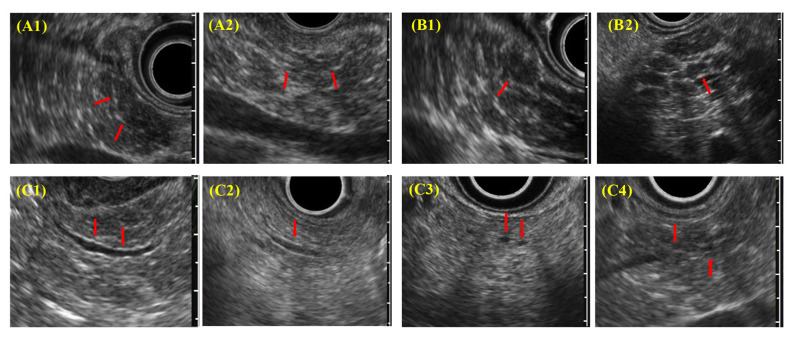
Each EUS finding in DCECP2019; (**A1**,**A2**) hyperechoic foci; nonshadowing/stranding; (**B1**,**B2**) lobularity (nonhoneycombing/honeycombing type); (**C1**,**C2**) hyperechoic MPD margins; (**C3**,**C4**) dilated side branches.

EUS diagnostic findings (Figure 3) include (A) hyperechoic foci with nonshadowing or stranding, (B) lobularity (nonhoneycombing or honeycombing type), (C) hyperechoic MPD margins, and (D) dilated side branches. ECP is diagnosed by imaging when two or more findings, including (A) or (B) or both, are observed. In addition, irregular dilatation in three or more branched pancreatic ducts, as seen on ERCP or magnetic resonance cholangiopancreatography, also served as imaging findings for ECP.

### 2.6. Equipment

The echoendoscope and universal ultrasound processors used were the GF-UE290, GF-UCT260, and EU-ME2 electronic scanners (Olympus Co., Tokyo, Japan).

### 2.7. Statistical Analysis

Data are shown as the mean ± standard deviation. Statistical analyses were performed using SPSS v. 27.0 statistical analysis software (SPSS Inc., Chicago, IL, USA). In this study, we used Fleiss’ K, which is a generalization of Scott’s pi (π) statistic, a statistical measure of inter-rater reliability [22,23]. This is also related to Cohen’s K statistic and Youden’s J statistic, which may be more appropriate in certain instances [24]. While Scott’s π and Cohen’s *K* are applicable to only two raters, Fleiss’ K works for any number of raters giving categorical ratings to a fixed number of items. The *K* statistic can be interpreted as expressing the extent to which the observed amount of agreement among raters exceeds what would be expected if all ratings were completely random. Notably, while Cohen’s K assumes that the same two raters have rated a set of items, when Fleiss’ kappa is used, although there is a fixed number of raters (e.g., three), different items may be rated by different individuals [22].

To evaluate IOR, the *K*-values were defined as follows: <0, no agreement; 0–0.20, slight; 0.21–0.40, fair; 0.41–0.60, moderate; 0.61–0.80, substantial; 0.81–1.00, almost perfect (Table S3) [25].

The statistics calculated in this study were evaluated by a statistician.

## 3. Results

### 3.1. Patient Characteristics

The baseline characteristics of the study population are shown in Table 1. Of the 100 consecutive patients with a median age of 64 years (±12 years), 63 (65%) were male and 27 (28%) had a history of AP. The reason for the EUS observation included alcohol abuse in 32 (36%), biliary stones in eight (8%), idiopathic causes in 47 (48%), and some other etiology in six (6%).

The positivity of each clinical finding in DCECP2009 and DCECP2019 was as follows: recurrent upper abdominal pain in 76 patients (78%), recurrent back pain in 27 patients (28%), abnormal pancreatic enzyme levels in the serum or urine in 36 patients (37%),abdominal pancreatic exocrine function in 0 patients (0%), pancreatitis-related susceptibility genes in 0 patients (0%), continuous heavy alcohol consumption equivalent to at least 80 g/day of pure EtOH in 18 patients (19%), and continuous heavy alcohol consumption equivalent to at least 60 g/day of pure EtOH in 32 patients (36%) (Table 1).

In DCECP2009, the number of cases per clinical finding was 0/1/2/3 (number of items) = 6/67/23/1 (number of cases). In contrast, in DCECP2019, the number of items was 0/1/2/3/4 (number of items) = 1/45/37/13/1 (number of cases).

### 3.2. IOR of Each EUS Finding in DCECP2009 and DCECP2019

The overall IOR for each EUS finding in DCECP2009 was as follows: (1) lobularity with honeycombing—*K* = 0.700, (2) lobularity without honeycombing—*K* = 0.661, (3) hyperechoic foci without shadowing—*K* = 0.570, (4) stranding—*K* = 0.524, (5) cysts—*K* = 0.816, (6) hyperechoic MPD margins—*K* = 0.771, and (7) dilated side branches—*K* = 0.552 (Table 2). The IOR for each EUS feature in DCECP2019 was as follows: (1) hyperechoic foci, nonshadowing/stranding—*K* = 0.741, (2) lobularity (nonhoneycombing/honeycombing type)—*K* = 0.706, (3) hyperechoic MPD margins—*K* = 0.771, and (4) dilated side branches—*K* = 0.552 (Table 3).

### 3.3. IOR of the DCECP2009 and DCECP2019 EUS Criteria

The overall K-value for the IOR of EUS criteria in DCECP2009 was 0.424 and was 0.563:0.231 for groups A:B. In DCECP2019, the *K*-value was 0.618 and was 0.733:0.442 for groups A:B (Table 4).

### 3.4. ECP Diagnosis between Pancreatologists and Nonpancreatologists

The ratio of ECP to normal cases according to the DCECP2009 EUS criteria was 77:72 cases, while that according to the DCECP2009 EUS criteria in group B was 72:25 cases. In contrast, the ratio of ECP to normal cases according to the DCECP2019 EUS criteria in group A was 49:48 cases, while that according to DCECP2019 in group B was 50:47 cases (Table 5).

### 3.5. Changes in the Final Diagnosis of ECP Based on Clinical and EUS Findings in DCECP2009 and DCECP2019

**Figure 4:**
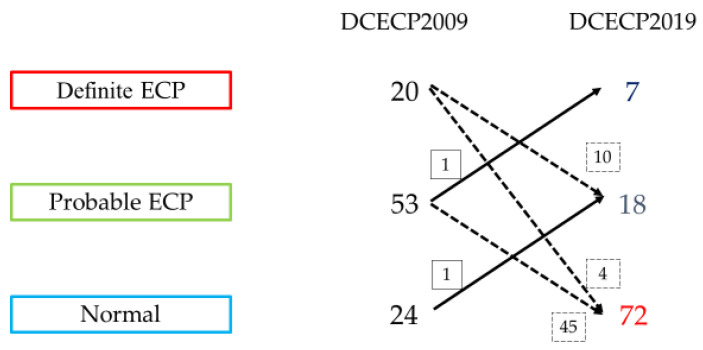
Changes in final diagnosis between DCECP2009 and DCECP2019, DCECP, diagnostic criteria for early chronic pancreatitis; ECP, early chronic pancreatitis.

In group A, *K* > 0.5 for both DCECP2009 and DCECP2019, and IOR was moderate. The EUS diagnostic findings in group A served as the final diagnosis. DCECP2009 included 20 cases of definite ECP, 53 cases of probable ECP, and 24 normal cases. In contrast, DCECP2019 included seven cases of definite ECP, 19 cases of probable ECP, and 71 normal cases (Figure 4).

## 4. Discussion

Recently, “ECP”, which describes the early stages of CP as opposed to later, more advanced stages, has been increasingly recognized. Research on the concept of ECP includes its early diagnosis and therapeutic intervention before CP becomes irreversible, which may aid in avoiding late-stage complication and improving clinical outcomes [26].

The first diagnostic criteria for ECP were introduced in the 2009 Japanese clinical diagnostic criteria for CP [27]. According to these criteria, ECP can be diagnosed based on clinical and imaging features. Particularly, EUS, which reveals specific features of ECP, was positioned as a very important imaging modality in DCECP2009, and the EUS findings adopted in these criteria were selected from the EUS diagnostic criteria (Rosemont classification) [21]. A previous report showed that approximately 5% of cases diagnosed as ECP according to the DCECP2009 have been shown to progress to definite CP, [6] and thus, the reliability of this diagnostic classification system has to some extent been accepted. However, the clinical features of ECP differ from those of definite and probable CP, such that ECP is associated with a high proportion of women, an idiopathic nature, and an older age at onset. In recent reports where DCECP2009 was used, the differences in the clinical features between CP and ECP posed a problem [6,7,28]. In addition, an international movement has begun to define CP mechanically, and a new definition of CP (a mechanistic definition) that incorporates the concept of ECP was proposed [3]. Consequently, the criteria for ECP were revised in 2019 (DCECP2019). Although the EUS criteria in DCECP2009 were established based on the Rosemont classification, it was reported that the Rosemont classification did not significantly increase IOR for the EUS-based diagnosis of CP compared with conventional scoring [18,29,30,31,32,33]. The reason for this is that it is difficult to interpret EUS findings of ECP. In DCECP2019, of the seven EUS features established in DCECP2009, the confounding features (hyperechoic foci and strands and lobularity with and without honeycomb) were grouped together into four categories. Thus, this revision is expected to simplify diagnosis and improve diagnostic specificity.

With this revision, if the diagnostic specificity of ECP increases, it is assumed that it would be useful for the early treatment intervention in patients with CP. However, these newly proposed criteria have never been validated or demonstrated to have better IOR than previous criteria. Furthermore, IOR of EUS findings for CP diagnosis is not always highly based on previous literature [18,19], and therefore, we performed this study to assess the significance of the new diagnostic criteria, DCECP2019, from the viewpoint of IOR of the EUS findings, which are crucial for diagnosis. In this study, K-values for each EUS finding were over 0.5 (moderate), and a high IOR was confirmed for each finding. Therefore, both sets of diagnostic criteria (DCECP2009 and DCECP2019), which were established according to EUS findings with high IOR, can be considered to have high reliability as diagnostic imaging features. Consequently, we compared the IOR of EUS criteria for an ECP diagnosis between DCECP2009 and DCECP2019. Our results indicated that the IOR of the DCECP2019 EUS criteria was higher than that of DCECP2009. This suggests that the reliability of the diagnostic criteria for ECP has been further improved in this revision.

In this study, we also investigated the IOR of DCECP by dividing the observers into pancreatologists and nonpancreatologists. The differences in IOR were associated with differences in specialty even among gastrointestinal endoscopy experts. In the pancreatologist group, *K* is >0.5 for both DCECP2009 and DCECP2019, which indicates moderate IOR of EUS criteria for both. However, the IOR of EUS criteria in the nonpancreatologist group was low for both criteria, which suggests that IOR of DCECP among nonpancreatologists is insufficient. Therefore, while the medical treatment of patients with advanced CP should be entrusted to a pancreatologist for early-stage CP diagnosis, various gastroenterologists should be involved in the diagnosis. Therefore, it is desirable to further educate all gastrointestinal endoscopists on the EUS diagnostic findings of ECP to promote and improve diagnosis.

We also examined how the diagnosis by pancreatologists based on DCECP2009 would change with DCECP2019. With the revision of the diagnostic items, the prevalence of definite and probable ECP and the prevalence of non-CP (normal) have increased.In DCECP2019, it is now possible to identify cases with risk factors for ECP by decreasing the alcohol consumption threshold and incorporating a history of AP in clinical diagnoses. On the contrary, the number of positive clinical findings increased from two to three, which suggests that DCECP2019 may be more suitable for identifying ECP patients with greater certainty than DCECP2009. This suggests that changes in both the ECP findings and the clinical diagnosis may have contributed to the change in final diagnosis. Therefore, the intended revision, DCECP2019 can better indicate “true” ECP.

The limitations of this study are the lack of a gold standard for diagnostic imaging of ECP, the single-center and retrospective design, the small number of patients, and that diagnosis being based on only three EUS images. However, this study only evaluated the IOR of each EUS finding for ECP and the diagnostic criteria, and thus, the lack of a gold standard is irrelevant. Nevertheless, a gold standard for ECP is still required to discuss the validity of these diagnostic criteria, and alternatively, positive follow-up may be necessary. Although the EUS was performed by various endosonographers, all cases were finalized/certified by experts. Therefore, although reliability of the EUS images is guaranteed, selection bias may be involved in the selection process. As a statistical limitation, the number of categories and subjects affects the magnitude of the value, and therefore, the K-value may be higher with fewer categories [34].

In the future, a prospective multicenter study that addresses these limitations will be needed.

## 5. Conclusions

IOR of the DCECP2019 EUS diagnostic criteria was higher than that of DCECP2009. Although the IOR of the EUS diagnostic criteria is considered a limitation of ECP diagnosis, our results suggest that DCECP2019 has improved precision and higher reliability than DCECP2009.

## Figures and Tables

**Table 1:** Patient characteristics and clinical features in DCECP2009 and DCECP2019.

Age, Mean ± SD (Range)	64 ± 12 (21–84)
Sex, male, *n* (%)	63 (65)
Reason for EUS observation, *n* (%)	
Alcohol abuse	32 (36)
Stone in biliary system	8 (8)
Recurrent upper abdominal pain, *n* (%)	76 (78)
Recurrent back pain, *n* (%)	27 (28)
Abnormal pancreatic enzyme levels in the serum or urine, *n* (%)	36 (37)
Abnormal pancreatic exocrine function, *n* (%)	0(0)
Pancreatitis-related susceptibility genes, *n* (%)	0(0)
Continuous heavy alcohol consumption equivalent to at least 80 g/day of pure ethanol, *n* (%)	18 (19)
Continuous heavy alcohol consumption equivalent to at least 60 g/day of pure ethanol, *n* (%)	32 (36)
History of AP, *n* (%)	27 (28)
Number of cases per clinical finding (number of items)	
DCECP2009 (0/1/2/3)	6/67/23/1
DCECP2019 (0/1/2/3/4)	1/45/37/13/1/

DCECP, diagnostic criteria for early chronic pancreatitis; SD, standard deviation; AP, acute pancreatitis.

**Table 2:** Interobserver reliability of each EUS finding in DCECP2009.

EUS Findings	Overall Kappa (95% CI)	A Group (Pancreatologists) Kappa (95% CI)	B Group (Nonpancreatologists) Kappa (95% CI)
Lobularity with honeycombing	0.700 (0.624–0.776)	0.736 (0.619–0.854)	0.583 (0.330–0.836)
Lobularity without honeycombing	0.661 (0.584–0.737)	0.730 (0.621–0.847)	0.467 (0.214–0.720)
Hyperechoic foci without shadowing	0.570 (0.494–0.647)	0.576 (0.459–0.693)	0.524 (0.271–0.777)
Stranding	0.524 (0.448–0.600)	0.545 (0.428–0.662)	0.457 (0.204–0.710)
Cysts	0.816 (0.740–0.893)	0.912 (0.795–1.029)	0.600 (0.347–0.853)
Dilated side branches	0.552 (0.476–0.628)	0.576 (0.459–0.693)	0.430 (0.177–0.683)
Hyperechoic MPD margins	0.771 (0.695–0.995)	0.812 (0.695–0.930)	0.734 (0.481–0.987)

MPD, main pancreatic duct.

**Table 3:** Interobserver reliability of each EUS finding in DCECP2019.

EUS Findings	Overall Kappa (95% CI)	A Group (Pancreatologists) Kappa (95% CI)	B Group (Nonpancreatologists) Kappa (95% CI)
Hyperechoic foci; nonshadowing/stranding	0.741 (0.687–0.795)	0.827 (0.744–0.910)	0.541 (0.362–0.720)
Lobularity	0.706 (0.652–0.760)	0.862 (0.779–0.945)	0.695 (0.516–0.874)
Hyperechoic MPD margins	0.771 (0.695–0.995)	0.812 (0.695–0.930)	0.734 (0.481–0.987)
Dilated side branches	0.552 (0.476–0.628)	0.576 (0.459–0.693)	0.430 (0.177–0.683)

MPD, main pancreatic duct.

**Table 4:** Interobserver reliability of EUS criteria in DCECP2009 and DCECP2019.

Observers	DCECP2009 Kappa (95% CI)	DCECP2019 Kappa (95% CI)
Overall	0.424 (0.399–0.448)	0.618 (0.594–0.643)
A group (pancreatologists)	0.563 (0.525–0.601)	0.733 (0.696–0.771)
B group (nonpancreatologists)	0.231 (0.150–0.313)	0.442 (0.361–0.523)

DCECP, diagnostic criteria for early chronic pancreatitis.

**Table 5:** Early chronic pancreatitis (ECP) diagnosis between pancreatologists and nonpancreatologists.

Observers	DCECP2009 ECP	Normal	DCECP2019 ECP	Normal
Overall	75	22	53	44
A group (pancreatologists)	77	20	49	48
B group (nonpancreatologists)	72	25	50	47

DCECP, diagnostic criteria for early chronic pancreatitis.

## Data Availability

No new data were created or analyzed in this study. Data sharing is not applicable to this article.

## Supplementary Materials

The following are available online at https://www.mdpi.com/2075-4418/11/3/431/s1: Supplementary Table S1. Clinical diagnostic criteria for early chronic pancreatitis 2009; Supplementary Table S2. Clinical diagnostic criteria for early chronic pancreatitis 2019; Supplementary Table S3. Interobserver reliability (K statistic).
