# Supplementary material for: Interobserver Reliability of the Endoscopic Ultrasound Criteria for the Diagnosis of Early Chronic Pancreatitis: Comparison between the 2009 and 2019 Japanese Diagnostic Criteria
Source: Diagnostics (Basel). 2021 Mar 3;11(3):431. doi: 10.3390/diagnostics11030431 (PMC8000630; doi:10.3390/diagnostics11030431)
Supplement: Supplementary file 1 [file diagnostics-11-00431-s001.pdf]

Supplementary Table 1. Clinical diagnostic criteria for early chronic pancreatitis 2009

<u>Clinical features</u>

(1) Repeated upper abdominal pain

(2) Abnormal pancreatic enzyme levels in the serum or urine

(3) Abnormal pancreatic exocrine function

(4) continuous heavy drinking of alcohol equivalent to or more than 80 g/day of pure ethanol (EtOH 80g/day)

<u>Imaging findings of early chronic pancreatitis (Either a or b)</u>

a. More than two features among the following seven features of EUS findings including at least one of (1)-(4)

(1) Lobularity with honeycombing

(2) Lobularity without honeycombing

(3) Hyperechoic foci without shadowing

(4) Stranding

(5) Cysts

(6) Dilated side branches

(7) Hyperechoic main pancreatic duct margin

b. Irregular dilatation of more than three duct branches on ERCP findings

Supplementary Table2. Clinical diagnostic criteria for early chronic pancreatitis 2019

Clinical features

(1) Repeated upper abdominal pain

(2) Abnormal pancreatic enzyme levels in the serum or urine

(3) Abnormal pancreatic exocrine function

(4) continuous heavy drinking of alcohol equivalent to or more than 60 g/day of pure ethanol (EtOH 60g/day) or pancreatitis-related susceptibility genes Continuous heavy drinking of alcohol

(5) Past-history of acute pancreatitis

Imaging findings of early chronic pancreatitis (Either a or b)

a. More than two features among the following four features of EUS findings including at least one of (1)-(2)

(1) Hyperechoic foci; non-shadowing/Stranding

(2) Lobularity [Nonhoneycombing/ honeycombing type]

(3) Hyperechoic main pancreatic duct margin

(4) Dilated side branches

b. Irregular dilatation of more than three duct branches on ERCP or MRCP findings

Supplementary Table 3. Interobserver reliability (K statics)

<0	No agreement
0.00-0.20	Slight
0.21-0.40	Fair
0.41-0.60	Moderate
0.61-0.80	Substantial
0.81-1.00	Almost perfect
